# Gut Microbiota Shifts in Pup Athymic BALB/c Mice: An Updated Identification in Nude Mice

**DOI:** 10.3390/ani9040151

**Published:** 2019-04-08

**Authors:** Yuting Li, Hao Sun, Beibei Du, Hui Xu

**Affiliations:** 1 Institute of Applied Ecology, Chinese Academy of Sciences, Shenyang 110016, China; liyuting211@mails.ucas.ac.cn (Y.L.); haos@spaces.ac.cn (H.S.); dubeibei16@mails.ucas.ac.cn (B.D.); 2 University of Chinese Academy of Sciences, Beijing 100049, China; 3 Key Laboratory of Pollution Ecology and Environmental Engineering, Institute of Applied Ecology, Chinese Academy of Sciences, Shenyang 110016, China

**Keywords:** athymic nude mice, gut microbiota, 16S rDNA sequencing

## Abstract

**Simple Summary:**

Mammal gut microbiota has been gradually considered to be related to innate and adaptive immunity. Incredibly, although athymic nude mouse is one of the most popular animals for modeling immunodeficiency and tumors, a basic understanding of its gut microbiota has still not been attained, and current relevant conclusions are controversial. In this 30-day study, based on high-throughput sequencing technology, we compared the differences in gut microbial community structures and functions between normal and nude pup mice, and concluded that gut microbiota shifts did occur in nude mice. These findings provide updated insight for the nude mouse tumor model.

**Abstract:**

It is commonly recognized that immunodeficiency modifies the gut microbiota in mammals. However, little information on the gut microbiota is available for athymic nude mice; one of the most popular animals for modeling immunodeficiency and tumors. In this study, 16S rDNA amplicon sequencing was performed to investigate the gut microbial composition of pup nude BALB/c mice during a 30-day experimental period. In contrast to pup normal mice, pup nude mice showed a significant variation in gut microbiota. Continuously decreased dynamics of the gut bacterial Shannon index, abnormal Firmicutes/Bacteroidetes ratio, the rarity of Bifidobacterium and Lactobacillus species, and a developmental lag of gut bacterial functions were observed in nude mice. The shift in gut microbiota and abnormal colonization of beneficial bacterial species in nude mice provide an updated insight into the nude mouse tumor model and a new perspective for establishing an animal model for study on dysbacteriosis.

## 1. Introduction

Billions of microbes populate the mammalian gut and are thought to influence host biology in many ways [1]. The establishment and development of the gut microbiota have been gradually considered to be related to innate and adaptive immunity, especially during early ontogeny [2]. Conversely, immunodeficiency in the early life of mammals may also lead to a shift in their gastrointestinal microbiota [3]. As immunodeficient animals, athymic nude mice represent the most popular and widely used in vivo tumor model [4,5]. Surprisingly, a basic understanding of the gut microbiota in nude mice has still not been attained, and current relevant conclusions are controversial [6].

Interactions between T cells and intestinal microbiota have been clearly demonstrated in numerous studies. In the intestines of mice, for example, the conversion of T_reg_ to TCRαβ^+^CD_4_^+^CD8αα^+^ T cells relies on the intestinal microbiota [7]; antimicrobial peptides expressed by CD8αβ^+^ intraepithelial lymphocytes result in the transplantation of *Bifidobacterium* [8]; commensal bacteria suppress retinoic acid synthesis by the intestinal epithelium, resulting in the control of Interleukin-22 (produced by T cells) activity [9]. In addition, Campbell and co-workers found that some bacterial species (e.g., *Mucispirillum schaedleri*) were lost in pT_reg_ cell-deficient mice [10].

The abovementioned studies impelled us to hypothesize that significant shifts in the gut microbiota should occur in congenital immunodeficient nude mice. However, the evidence to support this hypothesis is currently insufficient. Disappointingly, to the best of our knowledge, the only directly related study based on athymic nude mice did not support our hypothesis. In 1978, Brown et al. compared differences in gastrointestinal microflora between athymic and thymus-implanted BALB/c nude mice, and concluded that no dramatic difference occurred between these two groups [6]. However, due to limitations in the technologies and methods used at that time, the research only identified cultivable microbes, which account for only about 10% of the total microorganisms [11]. Based on current popular opinion, minor shifts in the gut microbiota cannot be precisely described by quantifying the changes in limited cultivable bacteria species. Therefore, we considered it necessary to update the understanding of gut microbes in athymic nude mice in a high-throughput sequencing technology-based study.

In this study, the dynamic changes in mouse gut microbiota from normal and nude pup BALB/c mice were investigated by 16S rDNA amplicon-based sequencing, aiming to verify whether significant gut microbiota shifts occur in congenital immunodeficient nude mice. 

## 2. Materials and Methods

### 2.1. Subjects and Sampling

Specific pathogen-free (SPF) normal BALB/c mice (B) and nude BALB/c mice (BN) (nine mice for each type, two-week-old weaning male pups) were purchased from China Medical University (Shenyang, China). Three mice were placed in each cage, and all mice were housed in the SPF-grade rooms and had free access to germ-free water and solid food.

The mice were acclimated to the new environment for one week. Afterwards, mouse stools were collected on day 1 (referred to as B1/BN1), day 14 (B14/BN14), or day 30 (B30/BN30), corresponding to mice aged 3, 5 or 8 weeks, respectively. In the morning of each sampling day, the mice were placed into sterile boxes, where they would defecate. Fresh stools were gathered from the cage and immediately weighed before performing DNA extraction.

All mice were not executed in this study, and the fecal collection did not bring any suffering to experimental mice. The experimental procedures complied with the guidelines approved by the Ethical Committee of Experimental Animal, China Medical University (Shenyang, China).

### 2.2. Sequencing and Reads Assembly

The QIAamp DNA Stool Mini Kit (Qiagen, Germany; catalogue #51504) was used to extract total DNA from the feces samples. The quantity of extracted DNA was determined with a Nano Drop 2000 instrument (Thermo, USA). PCR primers targeting the V3 and V4 regions of the bacterial 16S rDNA contained forward primers “CCTACGGRRBGCASCAGKVRVGAAT” and reverse primers “GGACTACNVGGGTWTCTAATCC”, which were designed by GENEWIZ, Inc. (Suzhou, China). All PCR reactions were performed with Phusion^®^ High-Fidelity PCR Master Mix (New England BioLabs, Ipswich, MA, USA).

Afterwards, all DNA samples were amplified in triplicate with no-template controls and detected by agarose electrophoresis. Three replicates of purified PCR amplicons were used to make sequencing libraries. All libraries were generated using the TruSeq^®^ DNA PCR-Free Sample Preparation Kit (Illumina, San Diego, CA, USA), following the manufacturer’s recommendations, and barcodes were added. Lastly, all libraries were sequenced on an Illumina MiSeq 300PE platform (Illumina, San Diego, CA, USA) at GENEWIZ, Inc. (Suzhou, China).

The sequencing reads were merged and quality filtered under specific filtering conditions, according to the Quantitative Insights Into Microbial Ecology (QIIME) 2 pipeline (QIIME™, V2.0) [12]. Chimeric sequences were identified using USEARCH software (V11.0, http://www.drive5.com/usearch/) and deleted. Sequences were assigned to each sample with a 12-base pair barcode using a QIIME 2 pipeline (QIIME™, V2.0). High-quality sequences from all samples were clustered into operational taxonomic units (OTUs) at 97% sequence similarity (OTU97) using the default QIIME pipeline and USEARCH’s UCLUST algorithm (http://www.drive5.com/usearch/). Representative sequences in each OTU97 were assigned to taxonomic groups using the Greengenes Database classifier with an 80% confidence threshold [13].

### 2.3. Statistical Analysis

Diversity indexes, and weighted and unweighted UniFrac (WUF and UUF) values were calculated with QIIME 2 (QIIME™, V2.0) and visualized with the “Phyloseq” package (Version 3.7) in R software (Version 3.4.3) [14]. Principal coordinate analysis (PCoA) was performed with the “Phyloseq” package in R software according to the WUF and UUF matrix. Top OTUs (phylum and genus levels) were counted and analyzed using GraphPad Prism 6 software (GraphPad Software Inc., San Diego, CA, USA). Phylotypic investigation of communities by reconstruction of unobserved states (PICRUSt) analysis was performed using the Galaxy online analysis platform (http://huttenhower.sph.harvard.edu/galaxy/). The results of the functional potential of bacterial assemblages were examined with PICRUSt, using level-2 and level-3 Kyoto Encyclopedia of Genes and Genomes (KEGG) orthologs. Statistical analyses of the KEGG orthologs were performed with the principal component analysis (PCA) module in Statistical Analysis of Metagenomic Profiles (STAMP) software (Version 2.1.3, Dalhousie University, Halifax, NS, Canada).

Linear discriminant analysis (LDA) effect size (LEfSe) analysis was performed to screen the differential taxa (phylum and genus) and differential bacterial functions between age-matched samples (B and BN), using an LDA score of 2.5 as a screening threshold. T-test analysis, using the Statistical Package for Social Sciences (SPSS) program (version 21.0, SPSS Inc., Chicago, IL, USA), was performed to explore the differences in diversity indexes and differentially abundant KEGG orthologs between age-matched samples. Pearson correlation analysis was performed using the SPSS program. Statistical comparisons of UniFrac matrix and KEGG orthologs among all samples were performed by permutational multivariate analysis of variance (PERMANOVA), using “Vegan” package in R software (Version 3.4.3) [14].

## 3. Results

### 3.1. Overview of 16S rDNA Sequencing Data

**Figure 1:**
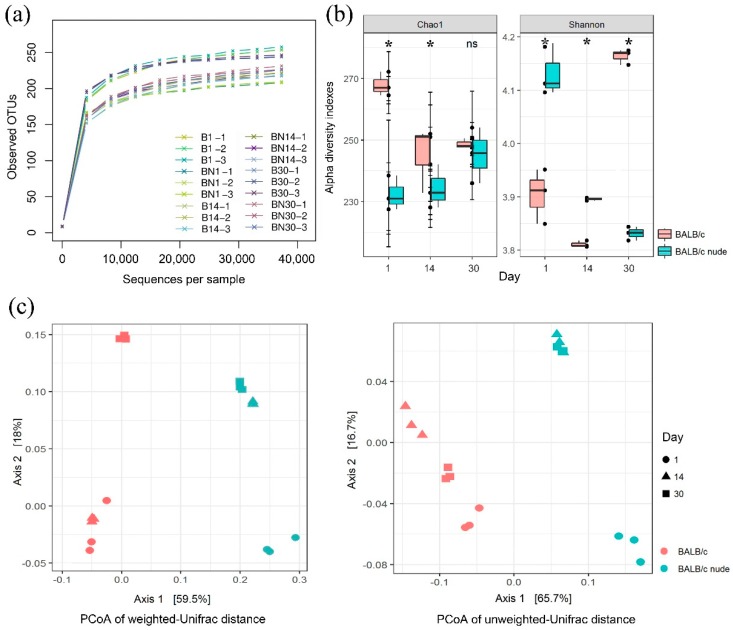
Gut microbial operational taxonomic units (OTUs) and diversity indexes during the development of normal and nude BALB/c pup mice. (**a**) Bacterial rarefaction curves based on observed OTUs were used to assess the depth of coverage for each sample. The samples are distinguished by different colors of lines. (**b**) Bacterial alpha diversity, as determined by the Chao1 and Shannon indexes. The asterisks show the significance determined by independent t-test, and “ns” indicates cases where the difference was not significant. (**c**) Scatterplots from principal coordinate analysis (PCoA), based on weighted and unweighted UniFrac distances of the OTUs in all samples at each of three time points.

A total of 1,349,980 sequences was detected from all samples. The number of sequences obtained from a single sample ranged from 54,119 to 90,520. Subsequently, 1,164,815 clean sequences were attained for downstream analysis (Supplementary Materials Table S1). Rarefaction curves demonstrated no new observed species (OBS) after 10,000 reads, which indicated that almost all bacterial species detected were observed in each sample (Figure 1a).

In general, significant differences observed in the number of observed species were found between age-matched samples in day 1 and day 30 (*p* < 0.001). The B1 samples showed the most bacterial species (average of 59 genera in nine phyla), while the BN1 samples showed the fewest bacterial species (average of 51 genera in eight phyla) (Supplementary Materials Table S1).

### 3.2. Variation in Gut Microbioal Diversities in Nude Mice

In normal pup BALB/c mice, the bacterial diversity (Shannon index) showed significant differences in the following order: B30 (4.16) > B1 (3.90) > B14 (3.81), while BN1 (4.14) > BN14 (3.90) > BN30 (3.83) in nude BALB/c mice (Figure 1b, Supplementary Materials Table S1, S2). Age-matched normal and nude mice showed significant differences in the Shannon index (Figure 1b). Mice in the BN samples had a higher Shannon index on the day 1 and day 14, when compared with age-matched mice in the B samples; however, B samples clearly showed a higher Shannon index on day 30 (Figure 1b).

Compared with the Shannon index, the dynamics of the Chao1 index manifested distinct trends. The Chao1 indexes ranged from high to low as follows: B1 (268) > B14 (250) > B30 (249) and BN30 (245) > BN14 (234) > BN1 (232) (Figure 1b, Supplementary Materials Table S1). Age-matched pup mice showed a conspicuous difference in the Chao1 index on day 1 and day 14 (Figure 1b); however, no statistical significances were found in nude mice samples among all sampling days (Supplementary Materials Table S2).

*T*-test and PERMANOVA were performed to investigate bacterial community variations in age-matched samples. On day 1, B1 and BN1 samples showed significantly different compositions of gut microbial communities (Supplementary Materials Table S2), and reversed results in terms of the Shannon and Chao1 indexes were also observed (Shannon: *p* = 0.006, Chao1: *p* = 0.001) (Figure 1b). On day 14, the diversity indexes in the two types of mice were considerably reduced, and the composition of the gut bacteria also displayed a statistically significant difference (Shannon: *p* = 0.000, Chao1: *p* = 0.034) (Figure 1b, Supplementary Materials Table S2). On day 30, B30 samples had the highest gut Shannon index, while the Shannon index of BN30 samples decreased dramatically (Shannon: *p* = 0.000, Chao1: *p* = 0.521) (Figure 1b, Supplementary Materials Table S2). From the perspective of bacterial indexes, the gut microbiota composition of normal mice on day 30 corroborated a previous report describing the gut composition of adult wild-type mice [15]. Over time, the normal pup mice recruited gut microbiota with enormous diversity, while nude mice lost their gut bacterial diversity.

UniFrac uses phylogenetic information to measure differences between two sequence clusters [16]. A relatively large UniFrac distance signifies that two communities are dissimilar, whereas smaller UniFrac distances signify more similar communities. The WUF (indicating relative abundance) and UUF (indicating presence or absence) results of age-matched B and BN samples showed that both the WUF and UUF distances on day 1 (namely B1 vs. BN1) were markedly higher than on day 14 and day 30, indicating that, compared with the older pup mice, the largest variation in gut microbiota between the age-matched samples was observed on day 1 (Supplementary Materials Figure S1).

UniFrac-coupled PCoA results illustrated the dynamics of gut bacterial communities on different sampling days. The bacterial communities of the B and BN samples generally separated into two groups along axis 1, which explained 65.7% and 59.5% of the variance in UUF and WUF, respectively (Figure 1c). These results clearly revealed that the B and BN samples divided into two separate clusters in axis 1, indicating that normal and nude mice retained their features within the time line. In other words, the composition of the gut bacteria in mice associated more on the type of mice than on their age. When comparing samples on different days, BN1 and B30 were relatively independent from other samples. Furthermore, PERMANOVA was performed to confirm the PCoA results (Supplementary Materials Table S2). WUF- and UUF-based microbiota distance metrics were significantly different between samples from different types of mice. Thus, it was demonstrated that the gut microbiota of pup BALB/c nude mice started to differentiate at a relatively younger age than occurred in pup normal mice.

### 3.3. Variation in Gut Microbial Composition of Nude Mice

**Figure 2:**
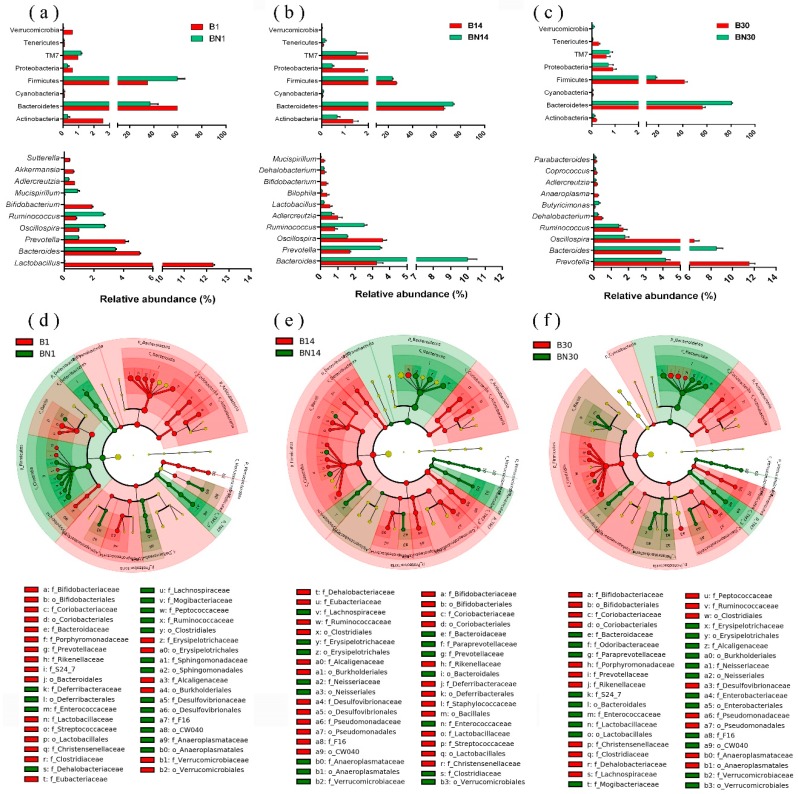
Variations of gut bacterial compositions in normal and nude BALB/c pup mice on days 1, 14 and 30. Panels (**a**–**c**) show the relative abundances of the most significant differentially abundant species (all phyla and top the 10 genera), as determined by linear effect size (LEfSe) analysis (linear discriminant analysis (LDA) score threshold > 2.5). Panels (**d**–**f**) show Cladogram plots representing the evolutionary branching of the differential bacterial species.

**Figure 3:**
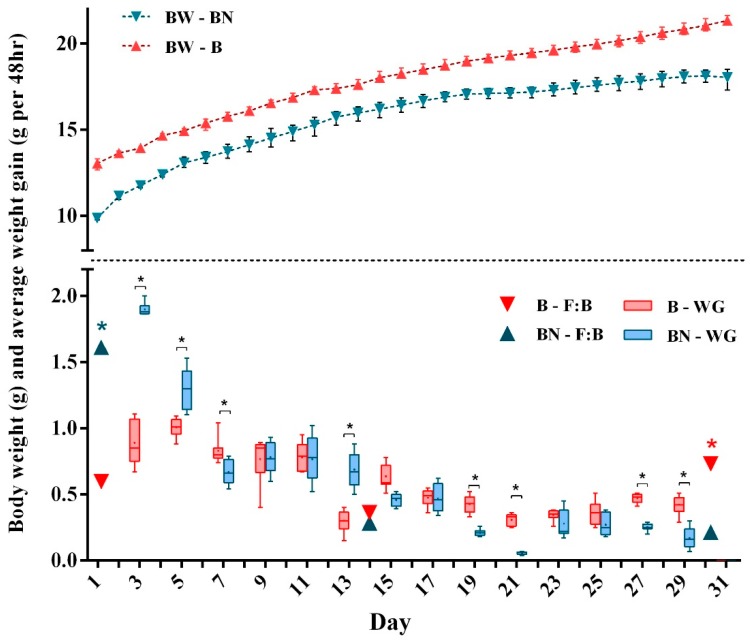
Body weight (BW), average body weight gain (BWG) and the Firmicutes:Bacteroidetes (F:B) ratio of normal and nude BALB/c pup mice. The asterisks show the significance determined by independent t-test (*p* < 0.05).

To fully identify the key members of the microbiota responsible for differences between the B and BN samples at the same sampling times, we used the LEfSe algorithm with statistical significance set at discriminant analysis scores >2.5 and *p* < 0.05 (Figure 2d–f), and LDA was performed to reveal distinct microbiota taxa at the phylum and genus levels (Figure 2a–c). At the phylum level, Bacteroidetes and Firmicutes were the dominating phyla of all samples. It is worth mentioning that age-matched samples showed totally different Firmicutes:Bacteroidetes (F:B) ratios on day 1, 0.59 in B1 and 1.61 in BN1. This finding suggested that Firmicutes strongly dominated the gut microbiota in pup nude mice on day 1. On the other two sampling days, the F:B ratios between the two types of mice (B14: 0.40, BN14: 0.32, B30: 0.74, BN30: 0.22) indicated that Bacteroidetes comprised a larger portion than Firmicutes (Figure 3, Supplementary Materials Table S3). Our study also showed that F:B ratios were solidly correlated to the average body weight gains (BWG). The most striking variation in BWGs between two groups was observed on the first sampling day, at which time BN samples showed a significantly higher (*p* < 0.05) BWG accompanied by a higher F:B ratio (Supplementary Materials Table S2). By day 14, both groups showed a downtrend in weight growth rate, simultaneously; no difference (*p* > 0.05) was observed in the F:B ratios between the two groups. In contrast to the results on the first sampling day, the BWG in the B30 samples was higher than that in BN30, and a higher F:B ratio was also observed in B30 (Figure 3).

At the genus level, we estimated the most differentially abundant and dominant genera (separately shown in Figure 2 and Supplementary Materials Table S4). On day 1, *Lactobacillus* (average predominance of 12.27% in B1) and *Roseburia* (average predominance of 9.10% in BN1) were the most abundant genera in B1 and BN1, respectively. Interestingly, the beneficial genus *Lactobacillus* rarely existed in BN1, which indicated that nude BALB/c mice might lose *Lactobacillus* at a very young age. Similarly, BN1 had a relatively low prevalence of several other important beneficial genera: *Akkermansia* (average abundance of 0.73% in B1 and 0.02% in BN1) and *Bifidobacterium* (average abundance of 2.11% in B1 and 0% in BN1) (Supplementary Materials Table S4).

On day 14, *Ruminococcaceae_UCG-014* (average predominance of 14.26%) and *Bacteroides* (average predominance of 9.75%) were the most abundant genera in the B and BN samples, respectively. Beneficial bacterial species (*Lactobacillus* and *Bifidobacterium*) abundant in the B1 samples were absent from the core genera in the B14 and BN14 samples (Supplementary Materials Figure S2). Nevertheless, LDA showed a remarkable difference in beneficial bacterial abundance between the two mouse types: The abundance of *Lactobacillus* averaged 0.50% in B14 and 0.20% in BN14, and that of *Bifidobacterium* averaged 0.35% in B14, and 0% in BN14. Apparently, normal mice were enriched with more beneficial bacteria on day 14 than nude mice.

The *Alistipes* (average predominance of 14.87%) and *Bacteroides* (average predominance of 8.55%) genera were the most abundant genera in the B30 and BN30 samples, respectively. LDA showed that as well as BN14, *Bacteroides* was the most dominant genera in BN30, when compared to B30 (averagely predominance of 8.55% while 3.78% in B30). *Butyricimonas*, considered as a butyrate-producing bacterium and a new probiotic for treating or relieving inflammatory bowel disease [17], was also relatively highly enriched in BN30 (average predominance of 0.23% in BN30 but only 0.06% in B30) (Figure 2c).

### 3.4. Functional Variation in Gut Microbiota in Nude Mice

**Figure 4:**
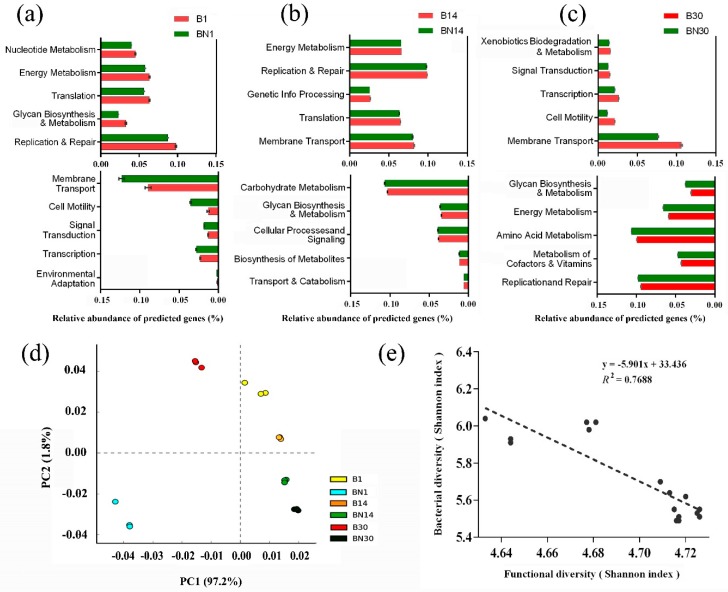
Variation in gut bacterial functional profiles in the pup normal and nude BALB/c mice at different time points. (**a**–**c**) The bar plots show the most significantly enriched differential bacterial genes in the two types of mice samples on day 1, day 14 and day 30, respectively. Significant differences are all obtained from displayed genes (determined by independent t-test, *p* < 0.05). (**d**) Regression analysis showed significant correlation between bacterial diversity and functional diversity in all samples. (**e**) The principal component analysis (PCA) plot indicated functional shifts in the gut microbial metagenome.

The PICRUSt results, which predicted the functional composition of the gut microbiomes, indicated that significant variations occurred in the gut microbial functions between two mouse types [18]. Functional profile data based on PERMANOVA analysis showed a significant difference in the age-matched samples at three different time points (Supplementary Materials Table S5). Regression analysis results (Figure 4c) illustrated a positive correlation (*r^2^* = 0.7688, *p* = 0.002) between the abundance of bacterial functional diversity and the bacterial diversity, indicating that variation in the gut bacterial functional profiles was driven by bacterial community shifts.

According to the functional profile data, most differential bacterial genes between time-matched samples were collected (*t*-test, *p* < 0.05). On the first observational day, normal mice enriched more genetic information processing-related genes (replication and repair, and translation) and metabolism-related genes (glycan biosynthesis and metabolism, energy metabolism, and nucleotide metabolism) than nude mice. While, nude mice significantly enriched environmental adaptation-, transcription-, signal transduction-, cell motility- and membrane transport-related genes (Figure 4a, Supplementary Materials Table S6).

On the day 14, the pup nude mice started to enrich more cellular processes- and metabolism-related bacterial genes (e.g., transport and catabolism, biosynthesis of other secondary metabolites, carbohydrate metabolism, and glycan biosynthesis and metabolism) which were all associated with bacterial proliferation, while the normal mice dominantly enriched more genetic information processing- and environmental information processing-related bacterial genes (e.g., energy metabolism, membrane transport, replication and repair, and translation) (Figure 4b). Exchanges of dominating bacterial genes were found in the two mouse types between day 1 and day 30 (namely B1 vs. BN30, BN1 vs. B30) (Figure 4a,c). Besides, the bacterial genes associated with xenobiotics biodegradation, and metabolism of cofactors and vitamins were respectively enriched in B30 and BN30 samples.

## 4. Discussion

In this study, the gut bacterial composition of pup BALB/c (wild-type/athymic) mice was revealed by 16S rDNA high-throughput sequencing. The results showed a significant difference in gut microbiota between the two types of mice during a 30-day observational period. Our findings were contrary to the only previous study, which suggested that the loss of T cell function does not dramatically alter the makeup of the cultivable gut microflora in the two mouse types [6]. Our study provides solid evidence that the reduction of T cells can cause gut microbiota shifts in pup BALB/c nude mice. In other words, BALB/c nude mice are incapable of establishing a gut microbiota resembling that in normal BALB/c.

Data from a previous study showed that the gut microbiota in human infants and animals present an increasing microbial diversity, over time scales from birth to the first year of life [19]. In this study, we found that over the course of 30 days, pup normal BALB/c mice showed markedly increased alpha diversity in the gut bacterial community, which was in accordance with a previous report [20]. In contrast, a decreasing variation was observed in nude mice throughout the 30-day period, based on the Shannon index (Figure 1b). The Shannon and Chao1 indexes of athymic BALB/c in this study indicate that, unlike healthy normal mice, pup nude mice cannot establish more homogeneous microbial structures with age. We also found the highest UniFrac distance on day 1 (B1 vs. BN1) (Supplementary Materials Figure S1), which confirmed that athymism-induced immunodeficiency was a powerful driving force of the gut microbiota composition of pup nude mice, especially at an early age. Additionally, in the cluster analysis (PCoA), a low distance was found between BN14 and BN30 samples (Figure 1c), indicating a high similarity in those samples. The findings above illustrated that the deletion of T cells affected the gut microbiota in athymic BALB/c mice at an early developmental period.

It is worth mentioning that a previous study with pTreg cell-deficient animals focused only on certain bacterial species (e.g., *Mucispirillum schaedleri*) [10], whereas, in our study, the overall gut microbiome shift in T cell-deficient animals was inspected from a more macro standpoint. In this study, the differential abundance of microbiota were observed at the phylum level in the two types of age-matched mice, which mainly showed a difference in the F:B ratio. The tremendously different F:B ratio between the B1 (0.59) and BN1 (1.61) samples implies that bacterial shifts in pup nude BALB/c mice lead to an abnormal occupation of excessive bacterial niches. The F:B ratio, a disease-related feature in the gut system, directly reflects the gut status of individuals [21], and the over-proliferation of Firmicutes is mainly responsible for the increased sugar absorption in the human body, which potentially gives rise to metabolic disorders, such as diabetes [22].

The highest abundance of Firmicutes observed in nude mice on day 1 indicates that the microbiota in BALB/c nude pup mice showed a functional trend towards higher nutrition utilization than that in normal mice. Coincidentally, nude mice on day 1 showed the highest BWG (Figure 3), which prompts us to consider is the relationship between the F:B ratio and BWG in the two types of mice. Then we further demonstrated, using the body weight data (Figure 3), that a high BWG is always accompanied with a high F:B ratio (*p* = 0.000, *r^2^* = 0.938), suggesting that the excessive proportion of Firmicutes improves the energy metabolism of pup BALB/c mice thus increasing their body weight.

Gut microbiota is a vital driving factor that affects energy harvest from the diet and energy storage in the host, which is also an additional contributing factor to weight gain [23]. Meanwhile, nude mice are defective in digesting function and nutrient metabolism [22]. Therefore, we speculate that high enrichment of Firmicutes in the BN group on day 1 may be related to a possible enteric dysfunction in pup nude mice, which might consequently promote nutrient absorption by some bacterial groups. In our study, dominant Firmicutes in pup nude mice indeed temporarily promoted their weight gain effectively; however, such promotion was not able to make up for the metabolic deficiency of nude mice during the observational period (Figure 3).

Pup nude mice tend to have lower proportions of bacterial species such as *Bifidobacterium* and *Lactobacillus* (Figure 2a–c, Supplementary Materials Figure S3), suggesting that these beneficial bacterial species may be incapable of adapting to the gut tract condition of nude mice. What we observed regarding beneficial genera in pup athymic mice contrasts with numerous comprehensive studies on healthy infants [20,24]. Our findings suggested that beneficial bacterial species existing in wild-type pup mice failed to occupy the gut bacterial niches of nude mice at an early stage (day 1). This outcome may be related to multiple possibilities, such as the compositions of milk nutritional components [25], immune globulins in breast milk [26,27], maternal gut microbiota [28], the vaginal and placental microbiota in the mother [29], and the deficiency of T cells in pup nude mice. Previous evidence indicates a firm link among gut microbiota, tumor characteristics, and host immunity in the tumor microenvironment [30]. Furthermore, accumulating evidence suggests that *Bifidobacteria* may enhance the antitumor immunity and efficacy of immunotherapy [30]. As secondary effects of immunodeficiency, the decrease in beneficial bacteria may also affect the development of cancer in BALB/c nude mice, which merits further attention. In addition, the decline of beneficial bacterial species in BALB/c nude mice provides the possibility of studying beneficial bacterial deletion in pups in a natural animal model, since animal models with dysbacteriosis are usually obtained through antibiotic treatment [31]. However, the side effects of antibiotics may interfere with related experiments. Therefore, BALB/c nude mice provide a new dysbacteriosis model without any additional pre-treatment. Therefore, we suggest that nude mice can be used not only for tumor modeling, but also for gut disturbance-related studies.

Along with the dynamics of gut microbiota during the 30 days, PICRUSt was applied to access the functional maturation of the gut microbiome in both types of mice. The nearest sequenced taxon index (NSTI) is used to evaluate the accuracy of PICRUSt predictions, and previous mammalian-associated microbiota samples showed a mean NSTI value of 0.14 ± 0.06 s.d. [32]. Thus, our mouse fecal samples, which had a mean NTSI value of 0.1412 ± 0.05 s.d., showed an ideal accuracy in terms of the PICRUSt prediction.

A functional data-based heat-map (Supplementary Materials Figure S2) indicates low abundance of most bacterial genes in nude mice on the first sampling day, suggesting that pup nude mice show an abnormal hysteretic in gut bacterial function compared with time-matched normal mice. Moreover, the PCA results imply a remarkable difference in gut microbial functions between normal and nude pup mice (Figure 4e). Besides, a far distance between B30 and BN30 were both shown by PCA analysis on the functional profile and PCoA analysis on the bacterial UniFrac matrix. Furthermore, a correlation between the bacterial and functional diversities in mice (Figure 4d) suggests that T cell-induced changes in the gut microbiota in pup nude mice can be significantly associated with functional shifts in the gut microbiota. All of the results above strongly suggest that immunodeficiency in pup nude mice caused by the absence of thymus result in the abnormal function of gut bacteria.

Bacterial reproduction-related genes (e.g., replication and repair, glycan biosynthesis and metabolism) were significantly enriched in B1 samples than BN1 samples (Figure 4a). Low enrichment of those genes in BN1 indicates that the gut bacteria in pup nude mice on day 1 cannot show effectiveness in DNA replication and bacterial cell wall biosynthesis, which will reduce their bacterial proliferation rate. Conversely, bacterial proliferation-related genes come to be significantly enriched in nude mice by day 14 (Figure 4b), which reconfirmed the developmental lag of gut microbiota in pup nude mice.

What is worth mentioning is that the significantly enriched bacterial genes in B1 are similar to BN30; BN1 and B30 also showed similarity (Figure 4a,c). The gut microbiota in nude mice enriches more propagation-related genes on day 30 than normal mice. This suggests that propagation decline is the fundamental flaw of the gut microbiota in nude mice during our observational period. Further, recent work has emphasized that host xenobiotic metabolism is shaped by the gut microbiome [33]. Xenobiotics biodegradation- and metabolism-related genes were uniquely significantly enriched in B30 (Figure 4c), which indicates normal mice can tackle complex organic compound metabolism by day 30. The decline of xenobiotic metabolism-related bacterial genes also suggests the weakness of gut bacterial function in nude mice.

Synchronously, the bacterial metabolism of cofactors and vitamins were dominantly enriched in nude mice (Figure 4c). We speculate that this may be related to immunodeficiency in nude mice. A model emerging from previous reference is that, during early mammalian development, the colonization of the host by commensal bacteria provides vitamin derivatives which can bind to an antigen-presenting protein that stimulates mobilizable immune cells, thus guarding against bacterial infections [34]. This suggests that nude mice may attempt to mobilize more immune cells in order to improve their anti-infective capacity by metabolizing more vitamins.

## 5. Conclusions

To the best of our knowledge, no sequencing-based study has been performed on the gut microbiota in BALB/c nude mice. The data generated in this study suggest new ideas regarding shifts in the gut microbial community in pup BALB/c nude mice. One significant finding was that significant differences occurred in the gut bacterial structure between normal and nude BALB/c pup mice, which was most obvious at an early stage. Together with the dynamics of the gut microbiota, functional shifts in the gut microflora in pup BALB/c nude mice were also triggered by T cell deficiency. Therefore, the impact of T cell immunodeficiency on gut microbes was far beyond our expectations. In addition, some important beneficial bacterial species showed a low abundance of colonization in pup athymic mice, especially at an early stage. These findings provide updated insight for the nude mouse tumor model and a new perspective on establishing an animal model of dysbacteriosis.

## Supplementary Materials

The following are available online at https://www.mdpi.com/2076-2615/9/4/151/s1, Table S1: General features of the high-throughput sequencing results, Table S2: Relative abundance (%) of Bacteroidetes and Firmicutes and ratio, Table S3: Comparisons of alpha indexes (Shannon and Chao1 indexes) and PERMANOVA analysis on (weighted and unweighted) UniFrac distances between samples, Table S4: Relative abundance (%) of the bacterial species in all samples, Table S5: PERMANOVA analysis of functional profile data, Table S6: Distribution of the predicted bacterial genes and relative abundance (%) in each sample type (KEGG, L2), Figure S1: Weighted (blue) and unweighted (red) UniFrac distances of age-matched normal and nude BALB/c pup mice on three sampling days. Different letters above the bars denote significantly different UniFrac distances among groups, Figure S2: Heat map of differentially abundant KEGG pathways (level-2) identified at three sampling time points (day 1, 14 and 30). The values of color in the heat map represent the normalized relative abundance, Figure S3: Relative abundance (%) of the *Bifidobacterium* and *Lactobacillus* in all samples.
