# Supplementary material for: Gut Microbiota Shifts in Pup Athymic BALB/c Mice: An Updated Identification in Nude Mice
Source: Animals (Basel). 2019 Apr 8;9(4):151. doi: 10.3390/ani9040151 (PMC6523615; doi:10.3390/ani9040151)
Supplement: Supplementary file 1 [file animals-09-00151-s001.pdf]

Gut Microbiota Shifts Are Found in Pup Athymic BALB/c Mice: Updated Identification in Nude Mice

Yuting Li [1,2], Hao Sun [1,3], Beibei Du [1,2] and Hui Xu [1,3]*

[1] Institute of Applied Ecology, Chinese Academy of Sciences, Shenyang 110016, China; liyuting211@mails.ucas.ac.cn (Y.L.); haos@spaces.ac.cn (H.S.); dubeibei16@mails.ucas.ac.cn (B.D.)
[2] University of Chinese Academy of Sciences, Beijing 100049, China
[3] Key Laboratory of Pollution Ecology and Environmental Engineering, Institute of Applied Ecology, Chinese Academy of Sciences, Shenyang 110016, China
* Correspondence: xuhui@iae.ac.cn; Tel.: +86-024-8397-0369

Supporting Information

Table S1. General features of the high-throughput sequencing results.

Sample ID	Raw Reads	Reads Analyzed	OBS OTUs	No. of Genera	No. of Family	No. of Order	No. of Class	No. of Phylum	Good's Coverage	Diversity Indexes	
										Chao1	Shannon
BN1-1	59723	51033	210	50	25	15	14	9	1	238.6	4.10
BN1-2	54119	45830	212	52	25	14	13	8	1	230.9	4.12
BN1-3	62197	52118	210	50	25	15	14	9	1	227.3	4.19
B1-1	77724	59172	255	59	25	15	14	9	1	264.6	3.84
B1-2	63820	48290	256	59	26	16	15	9	0.999	272.3	3.92
B1-3	61820	46106	259	59	26	15	14	9	1	266.8	3.95
BN14-1	90520	80860	223	54	27	16	14	9	1	242.3	3.90
BN14-2	81153	72969	219	55	27	16	14	9	1	228.2	3.89
BN14-3	75197	66970	219	53	27	16	14	9	1	232.7	3.91
B14-1	84838	76431	230	55	24	13	13	8	0.999	251.4	3.82
B14-2	91402	81102	225	53	25	14	14	8	0.999	252.3	3.81
B14-3	93199	82834	221	53	24	13	13	8	1	244.6	3.81
BN30-1	74921	69241	233	55	26	16	14	9	0.999	253.6	3.84
BN30-2	74980	69051	228	57	27	16	14	9	1	235.9	3.82
BN30-3	78944	72629	228	57	27	16	14	9	0.999	245.5	3.83
B30-1	67734	62765	245	58	25	15	14	8	1	247.3	4.17
B30-2	57988	53597	245	58	25	15	14	8	1	248.1	4.17
B30-3	99701	73817	247	59	26	16	14	8	1	250.6	4.15

Table S2. Comparisons of alpha indexes (Shannon and Chao1 indexes) and PERMANOVA analysis on (weighted and unweighted) UniFrac distances between samples.

Shannon index	df	P value	Mean1	Mean2	Diff.	SE of diff.	Sig.	t ratio
B1-vs-BN1	4	0.006	3.903	4.135	-0.232	0.043	**	-5.418
B14-vs-BN14	4	0.000	3.810	3.899	-0.089	0.005	***	-17.813
B30-vs-BN30	4	0.000	4.163	3.831	0.332	0.011	***	29.206
B1-vs-B14	4	0.049	3.903	3.810	0.093	0.033	*	2.805
B1-vs-B30	4	0.002	3.903	4.163	-0.260	0.034	**	-7.639
B14-vs-B30	4	0.000	3.810	4.163	-0.352	0.009	***	-39.175
BN1-vs-BN14	4	0.001	4.135	3.899	0.236	0.028	***	8.539
BN1-vs-BN30	4	0.000	4.135	3.831	0.304	0.028	***	10.733
BN14-vs-BN30	4	0.001	3.899	3.831	0.068	0.009	***	7.992
**Chao1 index**	**df**	**P value**	**Mean1**	**Mean2**	**Diff.**	**SE of diff.**	**Sig.**	**t ratio**
B1-vs-BN1	4	0.001	267.880	232.265	35.615	4.061	***	8.770
B14-vs-BN14	4	0.034	249.509	234.398	15.115	4.768	*	3.170

B30-vs-BN30	4	0.521	248.684	245.017	3.667	5.216	ns	0.703
B1-vs-B14	4	0.005	267.880	249.509	18.371	3.279	**	5.603
B1-vs-B30	4	0.002	267.880	248.684	19.167	2.493	**	7.699
B14-vs-B30	4	0.762	249.509	248.684	0.825	2.542	ns	0.325
BN1-vs-BN14	4	0.710	232.265	234.398	-2.128	5.337	ns	-0.399
BN1-vs-BN30	4	0.106	232.265	245.017	-12.751	6.123	ns	-2.083
BN14-vs-BN30	4	0.182	234.398	245.017	-10.623	6.594	ns	-1.611

PERMANOVA analysis on unweighted UniFrac matrix

	Df	Sums Of Sqs	Mean Sqs	F. Model	R^2	Pr (>F)	Sig.
**Samples**	2	3367.5	1683.73	194.24	0.95572	0.009	**
Residuals	18	156	8.67	0.04428			
Total	20	3523.5	1				
**Mouse Types**	6	3457.1	576.18	121.51	0.98116	0.036	*
Residuals	14	66.4	4.74	0.01884			
Total	20	3523.5	1				
**Date**	3	2565.5	855.18	15.176	0.72813	0.609	ns
Residuals	17	957.9	56.35	0.27187			
Total	20	3523.5	1				

PERMANOVA analysis on weighted UniFrac matrix

	Df	Sums Of Sqs	Mean Sqs	F. Model	R^2	Pr (>F)	Sig.
**Samples**	6	589.21	98.201	523.54	0.99556	0.001	***
Residuals	14	2.63	0.188	0.00444			
Total	20	591.83	1				
**Mouse Types**	2	582.16	291.078	541.36	0.98365	0.001	***
Residuals	18	9.68	0.538	0.01635			
Total	20	591.83	1				
**Date**	3	-1137.78	-379.26	-3.7277	-1.9225	0.906	ns
Residuals	17	1729.62	101.74	2.9225			
Total	20	591.83	1				

*, $p < 0.05$; **, $p < 0.01$; ***, $p < 0.001$.

Table S3. Relative abundance (%) of Bacteroidetes and Firmicutes and ratio.

BALB/c			BALB/c-nude		
**Sample ID**	**Bacteroidetes**	**Firmicutes**	**Sample ID**	**Bacteroidetes**	**Firmicutes**
B1-1	61	34	BN1-1	39	58
B1-2	61	34	BN1-2	39	59
B1-3	57	37	BN1-3	34	63
GRP B1	60	35	GRP BN1	37	60
**F:B ratio**	0.59		**F:B ratio**	1.61	
B14-1	66	27	BN14-1	74	23
B14-2	66	26	BN14-2	74	24
B14-3	66	27	BN14-3	74	23
GRP B14	66	27	GRP BN14	74	23
**F:B ratio**	0.40		**F:B ratio**	0.32	
B30-1	56	42	BN30-1	80	18
B30-2	56	42	BN30-2	81	17
B30-3	57	40	BN30-3	81	17
GRP B30	56	42	GRP BN30	81	18
**F:B ratio**	0.74		**F:B ratio**	0.22	

Table S4. Relative abundance (%) of the bacterial species in all samples. (Genus level, Top 10).

Genera Enriched	B1-1	B1-2	B1-3	Genera Enriched	BN1-1	BN1-2	BN1-3
Lactobacillus	13.22	11.59	11.99	*Roseburia*	8.84	9.04	9.42
Alistipes	6.64	7.51	8.57	*Lachnospiraceae*_NK4A136_group	5.91	5.82	7.76

*Ruminococcaceae*_UCG-014	5.10	5.38	5.60	*Lachnospiraceae*_UCG-001	5.59	5.96	5.75
Bacteroides	4.64	4.95	4.63	*Alistipes*	4.25	3.97	4.63
Roseburia	3.73	4.1	5.24	*Bacteroides*	3.46	3.62	3.25
*Prevotellaceae*_UCG-001	4.1	3.59	4.35	*Ruminiclostridium_5*	2.22	2.58	2.52
Bifidobacterium	2.09	1.98	2.25	*Lachnospiraceae*_FCS020_group	1.4	1.49	1.83
*Lachnospiraceae*_NK4A136_group	1.53	1.62	2.2	*Lachnospiraceae*_UCG-006	1.36	1.45	1.73
Alloprevotella	0.97	1.13	0.9	*Candidatus_Saccharimonas*	1.21	1.21	1.26
Candidatus_Saccharimonas	0.88	0.93	1.18	*Lachnoclostridium*	1.07	1.03	1.26
	**B14-1**	**B14-2**	**B14-3**		**BN14-1**	**BN14-2**	**BN14-3**
*Ruminococcaceae*_UCG-014	14.33	14.25	14.21	*Bacteroides*	9.88	9.45	9.91
Alistipes	6.98	7.12	7.07	*Ruminococcaceae*_UCG-014	9.70	9.90	9.63
Candidatus_Saccharimonas	3.46	4.12	3.92	*Prevotellaceae*_UCG-001	4.01	4.37	3.97
Bacteroides	3.09	3.24	2.98	*Alistipes*	4.08	4.08	3.94
*Lachnospiraceae*_NK4A136_group	2.22	2.21	2.62	*Alloprevotella*	3.46	3.5	3.36
*Prevotellaceae*_NK3B31_group	1.91	1.94	1.86	*Lachnospiraceae*_NK4A136_group	2.38	2.42	2.61
Alloprevotella	1.70	1.66	1.63	*Candidatus_Saccharimonas*	1.24	1.63	1.55
Pseudomonas	1.24	1.16	1.2	*Ruminiclostridium_5*	1.47	1.37	1.38
Enterorhabdus	0.96	0.78	0.97	*Rikenellaceae*_RC9_gut_group	1.20	1.23	1.20
*Prevotellaceae*_UCG-001	0.82	0.91	0.91	*Ruminiclostridium_6*	0.86	0.99	0.88
	**B30-1**	**B30-2**	**B30-3**		**BN30-1**	**BN30-2**	**BN30-3**
Alistipes	13.97	14.87	15.76	*Bacteroides*	8.31	8.64	8.70
Lachnospiraceae_NK4A136_group	11.86	11.83	11.6	*Alistipes*	6.18	6.34	6.72
Ruminococcaceae_UCG-014	10.46	10.41	9.94	*Ruminococcaceae*_UCG-014	4.71	4.72	4.71
Prevotellaceae_NK3B31_group	7.62	7.37	7.54	*Prevotellaceae*_UCG-001	3.99	4.28	4.37
Prevotellaceae_UCG-001	4.22	4.10	4.22	*Alloprevotella*	3.82	3.78	3.89
Bacteroides	3.76	3.85	3.74	*Rikenellaceae_RC9_gut_group*	2.30	2.26	2.36
Alloprevotella	3.01	2.98	2.91	*Lachnospiraceae_NK4A136_group*	2.07	1.80	1.80
Intestinimonas	0.86	0.83	0.78	*Ruminiclostridium_5*	0.86	0.85	0.86
Ruminiclostridium_5	0.78	0.83	0.80	*Candidatus_Saccharimonas*	0.75	0.76	0.73
Candidatus_Saccharimonas	0.70	0.49	0.64	*Roseburia*	0.57	0.51	0.56

Table S5. PERMANOVA analysis of functional profile data.

	Df	Sums Of Sqs	Mean Sqs	F. Model	R^2	Pr (>F)	Sig.
**Samples**	5	0.24609	0.049218	10.261	0.81044	0.002	**
Residuals	12	0.05756	0.004797	0.18956			
Total	17	0.30365	1				
**Mouse types**	1	0.083202	0.083202	6.0387	0.274	0.028	*
Residuals	16	0.22045	0.013778	0.726			
Total	17	0.303652	1				

*, $p < 0.05$; **, $p < 0.01$; ***, $p < 0.001$.

Table S6. Distribution of the predicted bacterial genes and relative abundance (%) in each sample type (KEGG, L2).

	B1	B14	B30	BN1	BN14	BN30
Cellular Processes,Cell Communication	0.000	0.000	0.000	0.000	0.000	0.000
Cellular Processes,Cell Growth and Death	0.583	0.583	0.549	0.511	0.577	0.582
Cellular Processes,Cell Motility	1.283	1.215	2.125	3.555	1.211	1.161
Cellular Processes,Transport and Catabolism	0.519	0.558	0.465	0.345	0.588	0.616
Environmental Information Processing,Membrane Transport	8.932	8.203	10.606	12.342	8.024	7.658
Environmental Information Processing,Signal Transduction	1.307	1.309	1.511	1.854	1.266	1.276
Environmental Information Processing,Signaling Molecules & Interaction	0.218	0.201	0.195	0.181	0.208	0.212
Genetic Information Processing,Folding, Sorting and Degradation	2.722	2.795	2.513	2.334	2.768	2.801
Genetic Information Processing,Replication and Repair	9.775	9.879	9.428	8.727	9.806	9.792
Genetic Information Processing,Transcription	2.315	2.189	2.641	2.818	2.203	2.125
Genetic Information Processing,Translation	6.338	6.485	6.016	5.609	6.346	6.325
Human Diseases,Cancers	0.100	0.106	0.108	0.093	0.109	0.110
Human Diseases,Cardiovascular Diseases	0.000	0.000	0.000	0.000	0.000	0.000
Human Diseases,Immune System Diseases	0.054	0.043	0.041	0.043	0.048	0.050
Human Diseases,Infectious Diseases	0.379	0.369	0.354	0.332	0.374	0.382
Human Diseases,Metabolic Diseases	0.136	0.143	0.123	0.104	0.144	0.145
Human Diseases,Neurodegenerative Diseases	0.136	0.146	0.134	0.110	0.138	0.144
Metabolism,Amino Acid Metabolism	10.245	10.625	9.937	10.792	10.558	10.623
Metabolism,Biosynthesis of Other Secondary Metabolites	1.097	1.121	1.088	0.954	1.170	1.208

Metabolism,Carbohydrate Metabolism	10.598	10.315	10.271	13.060	10.664	10.571
Metabolism,Energy Metabolism	6.348	6.602	5.887	8.485	6.532	6.575
Metabolism,Enzyme Families	2.249	2.245	2.264	2.158	2.268	2.288
Metabolism,Glycan Biosynthesis and Metabolism	3.276	3.428	3.020	2.280	3.578	3.749
Metabolism,Lipid Metabolism	2.869	2.795	2.735	2.812	2.809	2.792
Metabolism,Metabolism of Cofactors and Vitamins	4.498	4.637	4.287	5.120	4.603	4.720
Metabolism,Metabolism of Other Amino Acids	1.647	1.657	1.578	1.519	1.663	1.701
Metabolism,Metabolism of Terpenoids and Polyketides	1.783	1.831	1.646	1.539	1.805	1.812
Metabolism,Nucleotide Metabolism	4.515	4.503	4.293	3.987	4.471	4.498
Metabolism,Xenobiotics Biodegradation and Metabolism	1.591	1.471	1.456	1.866	1.452	1.445
Organismal Systems,Circulatory System	0.000	0.002	0.001	0.000	0.000	0.000
Organismal Systems,Digestive System	0.047	0.046	0.046	0.020	0.056	0.057
Organismal Systems,Endocrine System	0.319	0.333	0.328	0.260	0.350	0.348
Organismal Systems,Environmental Adaptation	0.140	0.139	0.154	0.201	0.137	0.133
Organismal Systems,Excretory System	0.035	0.039	0.031	0.016	0.040	0.043
Organismal Systems,Immune System	0.098	0.102	0.101	0.089	0.106	0.106
Organismal Systems,Nervous System	0.108	0.115	0.110	0.099	0.114	0.113
Organismal Systems,Sensory System	0.000	0.000	0.000	0.000	0.000	0.000
Unclassified,Cellular Processes and Signaling	3.798	3.808	3.821	3.799	3.892	3.915
Unclassified,Genetic Information Processing	2.596	2.626	2.588	2.484	2.549	2.535
Unclassified,Metabolism	2.570	2.526	2.599	2.341	2.554	2.572
Unclassified,Poorly Characterized	4.773	4.810	4.854	4.669	4.818	4.836

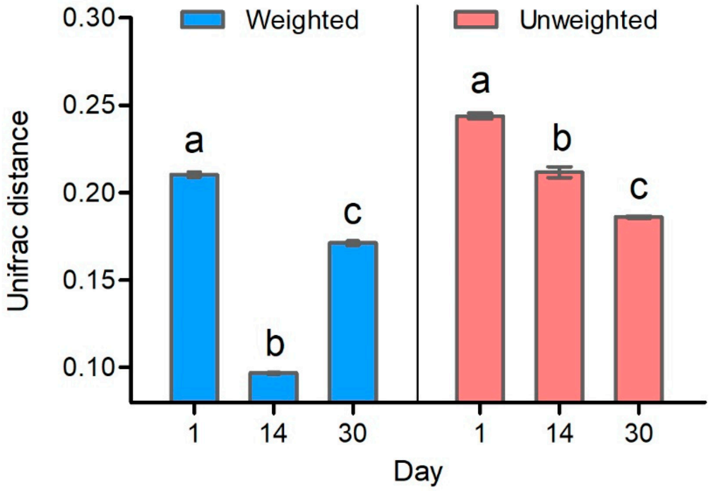

Figure S1. Weighted (blue) and unweighted (red) UniFrac distances of age-matched normal and nude BALB/c pup mice on three sampling days. Different letters above the bars denote significantly differentially UniFrac distance among groups.

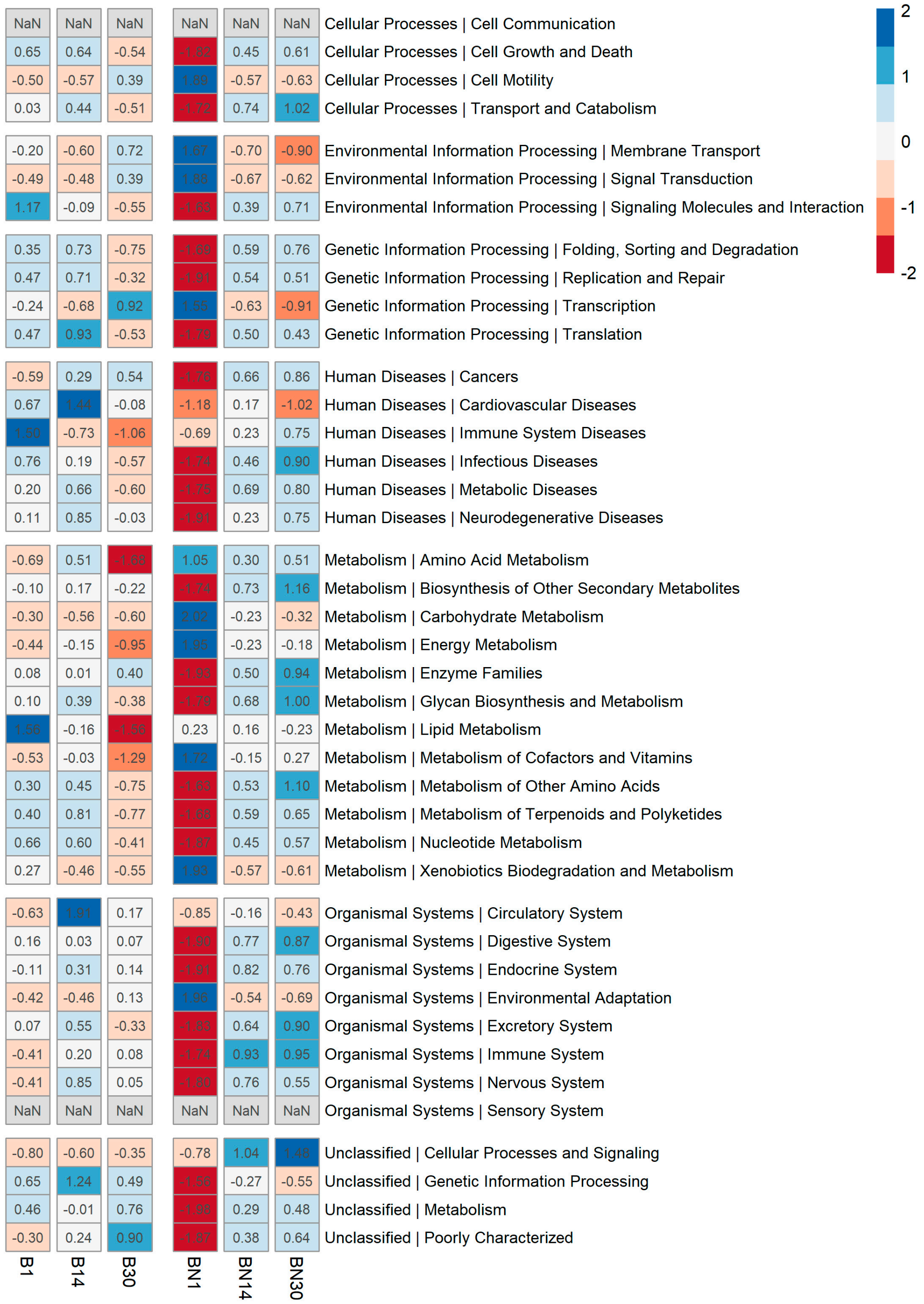

Figure S2. Heat map of differentially abundant KEGG pathways (level-2) identified at 3 sampling time points (day 1, 14 and 30). The values of colour in the heat map represent the normalized relative abundance (log10).

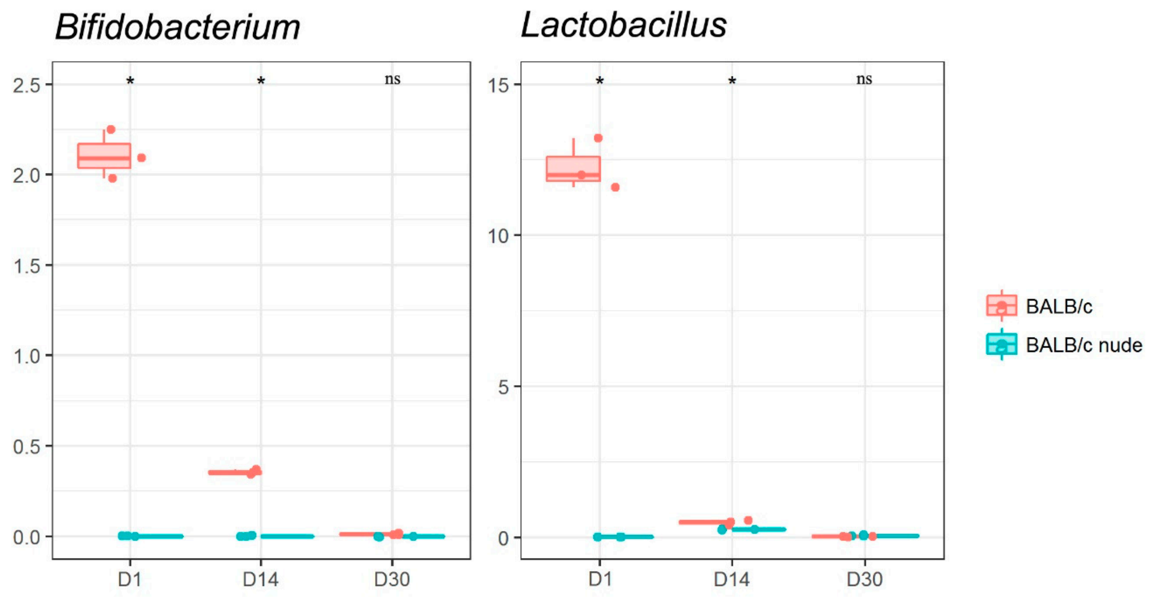

Figure S3. Relative abundance (%) of the *Bifidobacterium* and *Lactobacillus* in all samples.
